# Patients with Hypocortisolism Treated with Continuous Subcutaneous Hydrocortisone Infusion (CSHI): An Option for Poorly Controlled Patients

**DOI:** 10.1155/2023/5315059

**Published:** 2023-03-20

**Authors:** Malene Lyder Mortensen, Marie Juul Ornstrup, Claus H. Gravholt

**Affiliations:** ^1^Department of Endocrinology and Internal Medicine, Aarhus University Hospital, Aarhus, Denmark; ^2^Department of Molecular Medicine, Aarhus University Hospital, Aarhus, Denmark; ^3^Department of Clinical Medicine, Aarhus University, Aarhus, Denmark

## Abstract

**Objective:**

Despite appropriate oral glucocorticoid replacement therapy, patients with hypocortisolism often suffer from impaired health and frequent hospitalizations. Continuous subcutaneous hydrocortisone infusion (CSHI) has been developed as an attempt to improve the health status of these patients. The objective of this study was to compare the effects of CSHI to conventional oral treatment on hospitalizations, glucocorticoid doses, and subjective health status. *Patients*. Nine Danish patients (males: 4 and females: 5) with adrenal insufficiency (AI) were included, with a median age of 48 years, due to Addison (*n* = 4), congenital adrenal hyperplasia (*n* = 1), steroid induced secondary adrenal insufficiency (*n* = 2), morphine induced secondary adrenal insufficiency (*n* = 1), and Sheehan's syndrome (*n* = 1). Only patients with severe symptoms of cortisol deficit on oral treatment were selected for CSHI. Their usual oral hydrocortisone doses varied from 25–80 mg per day. The duration of follow-up depended on when the treatment was changed. The first patient started CSHI in 2009 and the last in 2021.

**Design:**

A retrospective case series comparing hospitalizations and glucocorticoid doses before and after treatment with CSHI. In addition, patients were retrospectively interviewed about their health-related quality of life (HRQoL) after the change of treatment modality.

**Results:**

Patients significantly reduced their daily dose of glucocorticoids by 16.1 mg (*p* = 0.02) after changing to CSHI. The number of hospital admission due to adrenal crisis decreased by 1.3 per year on CSHI, which was a 50% reduction (*p* = 0.04). All patients found it easier to handle an adrenal crisis with CSHI, and almost all patients found it easier to overcome everyday activities and had fewer symptoms of cortisol deficit such as abdominal pain and nausea (7-8 out of 9 patients).

**Conclusions:**

The change of treatment from conventional oral hydrocortisone to CSHI resulted in a reduced daily dose of glucocorticoids and a reduced number of hospitalizations. Patients reported regain of energy, achievement of better disease control, and better handling of adrenal crisis.

## 1. Introduction

Patients with adrenal insufficiency (AI) have inadequate production of cortisol [1]. The condition requires life-long glucocorticoid (GC) replacement therapy and stress adaptation to prevent adrenal crisis. Despite adequate treatment, patients with AI have an increased mortality risk and report fatigue in addition to reduced quality of life (QoL), questioning the quality of current treatment regimens [2–5]. The conventional replacement therapy shows heterogeneity across Europe, but the most frequently used is oral hydrocortisone (OHC) administered twice or thrice daily, with the highest dose administered in the morning [6]. Under normal physiological conditions, the circulating levels of cortisol follow a distinct circadian rhythm with the lowest concentration in the evening and during the night, replaced by rising levels in the early morning. Around awakening, a peak in the cortisol level appears, followed by declining concentrations during the daytime [7]. The impaired health and QoL in patients with AI may reflect that the conventional OHC replacement therapy fails to mimic this physiological pattern of cortisol secretion. In fact, treatment with OHC often leads to alternately unphysiological high or low levels of circulating GC throughout the day, and the average patient on conventional treatment receives a daily dose of GC that exceeds the expected need [8]. Excessive exposure to GC is associated with many adverse effects including hypertension, obesity, osteoporosis, and neuropsychiatric symptoms [9, 10]. An alternative and novel treatment possibility of AI is continuous subcutaneous hydrocortisone infusion (CSHI), which has been able to imitate the physiological cortisol circadian rhythmicity better than oral treatment [11–13]. The treatment with CSHI has so far been reserved for special cases due to a lack of scientific evidence and the complexity of living with a pump, in addition to the high price of pumps and inaccessibility to getting a pump.

At present, there is a paucity of studies investigating the treatment of AI with CSHI. Here, we report the experience of 9 Danish patients with AI treated with CSHI, which contributes to the current understanding of the possibilities CSHI provides in treating AI. We report patient's experiences with CSHI and investigate the effect on daily GC dose, body mass index (BMI), hospital admissions, and HRQoL.

## 2. Materials and Methods

Nine patients with different forms of AI were treated with CSHI based on clinical evaluation at Aarhus University Hospital, Denmark. Patients were referred from hospitals in all parts of Denmark, and only patients who suffered from severe symptoms of cortisol deficit on standard OHC treatment were selected for CSHI and are presented in this case series (Table 1).

In February 2022, medical records were reviewed to gather information about hospitalizations and GC dose before and after treatment with CSHI. Hospitalizations were counted as admissions per year, starting from the patients' first admission due to AI. All hospital admissions were included except elective procedures, and they were divided into 3 groups: emergency department visits, hospital admissions due to adrenal crisis, and hospital admissions without an adrenal crisis (Supplementary information 1). Hospital admissions with adrenal crisis were defined as admissions due to symptoms of adrenal crisis such as nausea, vomiting, and abdominal pain, and/or where patients were treated with intravenous GCs (see further details in Supplementary information 1). Patients were retrospectively interviewed about their experiences with the change of treatment regimen. Questionnaires were developed with inspiration from AddiQoL, which is an Addison-specific health-related questionnaire [14]. The questionnaire in this study consists of 20 questions with different topics including fatigue, psychological aspects, AI-symptoms, sleep, and everyday life (Supplementary information 2). The questionnaire was used to evaluate how the CSHI treatment affected QoL and the subjective well-being of the patients. All patients were asked to focus their question response on before and after the change of treatment.

Prior to the CSHI treatment, all patients were treated with oral GC replacement therapy with different regimens. Four patients received hydrocortisone thrice a day with the largest dose administered in the morning. Five patients were treated with Plenadren in the morning, a once-daily dose with a dual-release system, however, also supplemented with hydrocortisone in the afternoon (Supplementary information 3).

For CSHI we used hydrocortisone (Solu-Cortef) of 100 mg/2 ml solution in an insulin pump (model 740G, Medtronic). The pump had a basal rate setting, providing the patients with a basal daily dose of hydrocortisone. Basal infusion rates were calculated with inspiration from the feasibility study of Løvås and Husebye [11]. To determine the initial total daily dose, we considered the body weight of the patient, the previous oral dose of hydrocortisone, and a thorough discussion with the patient concerning the need for GC in their current situation. Infusion rates were divided into time slots, and the main principle was to provide cortisol in a physiological pattern with the highest doses around awakening and decreasing doses throughout the day. The individual doses were continuously adjusted based on clinical symptomatology to reach the lowest possible dose. The pump also allowed bolus infusions, which made it possible for patients to get extra cortisol when needed. Patients were educated to self-manage the pump, including knowledge on how to manage pump failure, how to change the cannulas, how to self-administer bolus doses in case of fever or other stress situations, and sick days.

## 3. Statistics

Changes in GC doses, hospital admissions, and BMI before and after the introduction of CSHI were analyzed by paired *t-* test in Excel and considered significant at a level of *p* < 0.05. We present data with median and interquartile range, or mean and standard deviation, as appropriate, depending on normality or non-normality distribution of data.

### 3.1. Ethics

The case series was performed as part of an evaluation of CSHI and was approved by the hospital review board. All patients consented to participate in this study.

## 4. Results

**Figure 1:**
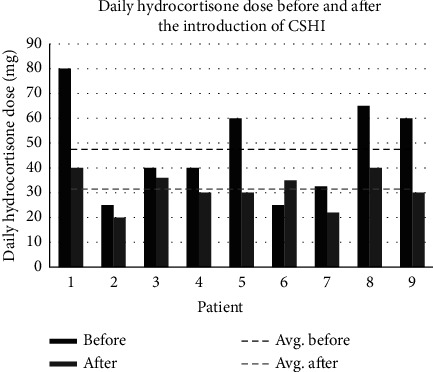
Daily hydrocortisone dose before and after the introduction of CSHI. CSHI:  continuous subcutaneous hydrocortisone infusions.

The patients in this case series were men and women aged between 36 and 58 years (Table 1). All patients were diagnosed with AI, and most of them had additional comorbidity including type 1 diabetes, ulcerative colitis, and chronic pain conditions. They had been treated with CSHI for varying periods of time. The first patient started CSHI in 2009 and the last patient in September 2021. On average, the number of hospital admissions due to adrenal crisis decreased by 50% after switching from conventional treatment to the CSHI regimen (from 2.6 hospital admissions per year to 1.3 hospital admissions per year, *p* = 0.04) (Table 2). Four patients had not been hospitalized in the years around the change of treatment. The other 5 patients had all reduced their number of hospital admissions after switching to CSHI. Patients 5 and 8 had been hospitalized with adrenal crisis before CSHI was initiated, but neither have had any hospitalizations with an adrenal crisis after the change of treatment to CSHI. Eight out of nine patients received a lower daily dose of GCs with CSHI compared to OHC treatment (Figure 1). The average daily dose equivalent during conventional treatment was 47.5 mg hydrocortisone, which was significantly reduced by 34% to 31.4 mg hydrocortisone on CSHI (*p* = 0.02).

Five out of eight patients lost weight after changing to CSHI (Table 3), however, the average BMI did not change (*p*=0.4). One patient was not included because of missing information.

### 4.1. Subjective Health Status

All patients found it easier to handle symptoms of adrenal crisis after changing to CSHI, and they all preferred CSHI over standard oral treatment (Table 4). In addition, most found it easier to overcome daily activities, experienced a positive mood change, and had less abdominal pain and nausea (8 out of 9 patients). Furthermore, most felt more rested in the morning and more energetic during the day (7 out of 9). Most experienced a positive effect on their cognitive function (5 out of 9), while potential symptoms of GC deficit, such as muscle pain and headache, were reduced in three and four patients, respectively, however not all patients experienced those symptoms before initiating CSHI. Some experienced better quality of sleep and surplus of energy for social activities (4 out of 9), and an increased sex drive (4 out of 9), while none reported fewer infections. When considering the potential downsides of having a CSHI system, six of nine patients found CSHI more troublesome than conventional oral treatment, and two of nine reported psychological problems with regard to wearing the pump. Finally, all patients were asked to grade their general well-being; “How are you today, compared to before you got the pump, on a scale from 1 to 10”? One represents “miserable” and 10 represents “great.” All patients experienced considerable and significant improvements regarding their overall well-being (Table 5). They graded themselves with very low well-being while on conventional OHC (range 1–4), with a marked increase after switching to CSHI (range 4–10), with a total average change of +6.8 (*p* < 0.001) (Table 5).

## 5. Discussion

In this case series, we present nine patients with hypocortisolism selected for treatment with CSHI due to very poor disease-control on standard OHC. The patients generally received high doses of OHC before switching to CSHI, with an average dose equaling 47.5 mg hydrocortisone per day, and some of these were treated with doses as high as 80 mg OHC per day. It is common that patients treated with OHC often receive too high doses of GCs, but conversely have an insufficient cortisol coverage when higher doses are needed during infections and stressful situations [8, 15]. In adults with Addison's disease (AD), the recommended daily dosage of hydrocortisone is approximately 15–25 mg per day [16]; thus, our study cohort was grossly overtreated with supraphysiological doses of OHC at baseline, and the high dose was actually one of the reasons for switching the patients to CSHI. Chronic excessive GC treatment has many adverse side effects such as osteoporosis, gain of weight, infections, increased risk of the metabolic syndrome, and Cushing's syndrome [9, 15]. When compared to the general population, patients with AD have a 2-fold higher mortality [17]. It is reasonable to suggest that the reduced subjective health status and the higher mortality in patients with AI may relate to long-term treatment with supraphysiological doses of GCs, and undoubtedly, a reduction in GC dose could be beneficial for the patients [17]. Patients in the present study significantly reduced their daily dose of hydrocortisone by 34%, corresponding to a daily dose reduction of 16.1 mg hydrocortisone after changing to CSHI; however, many still received a dose that is higher than recommended for which we have no ready explanations. Several other case series also found that patients were able to reduce their GC dose with CSHI. In 2007 Løvås and Husebye [11] did a pilot study aiming to investigate the technical feasibility, tolerability, and safety of CSHI. They treated 7 patients with AD with CSHI for up to three months and found that patients were able to reduce their GC dose by 40–85%. Khanna et al. [18] treated 3 AI patients with CSHI for an average of 17 months. Patients in this study reduced the daily dose of hydrocortisone by 14% on average compared to the previous OHC dose. Cardini et al. [19] used CSHI to treat a 42-year-old woman with secondary AI. After 14 months, the steroid dose was lowered by 15% and in addition to this beneficial effect, an AI-specific quality-of-life questionnaire (AddiQoL) score also improved.

Standard OHC replacement treatment fails to mimic the physiological circadian rhythm of circulating cortisol levels, and this may also be part of the explanation for why patients with AI suffer from fatigue, reduced quality of life, and impaired health, especially after many years of AI [20, 21]. Our data show that all 9 patients in the current study felt much better on CSHI than on standard OHC treatment, with a greater ability to overcome everyday activities and the feeling of having more energy during the day. However, there is a risk of recall bias, since the evaluation was performed retrospectively. A problem with OHC replacement therapy is that the early morning rise in cortisol levels seen in healthy individuals is absent [22]. The lack of cortisol peak in the morning may explain why patients wake up feeling unrested and unable to do anything before several hours after awakening. Six out of nine patients in the present audit felt more rested in the morning when receiving CSHI treatment, on which we know the circadian rhythm of cortisol is restored, including the early morning peak. Björnsdottir et al. [13] conducted a randomized crossover study and compared insulin sensitivity and glucose levels under CSHI versus OHC in 15 patients. All patients completed 8 weeks in each arm of the study. Insulin sensitivity was evaluated by a euglycaemic-hyperinsulinaemic clamp, a method used to quantify insulin resistance. Nighttime glucose levels were more stable in patients receiving CSHI compared to OHC, without compromising insulin sensitivity. In addition, CSHI provided a more physiological circadian cortisol pattern. Løvås and Husebye [11] reported 24-hour salivary and serum cortisol profiles that proved CSHI capable of re-establishing the circadian rhythm of cortisol levels. Our study is not the first to document how a change of treatment from OHC to CSHI improves subjective well-being in patients with AI. Nella et al. [23] compared CSHI to conventional OHC treatment in 8 patients with congenital adrenal hyperplasia. The treatment was evaluated by SF-36 Vitality score, AddiQoL, and a fatigue questionnaire, in addition to 24-hour hormonal samplings. After 6 months of follow-up, CSHI had positively affected HRQoL scores, as well as adrenal steroid control. Six patients chose to continue long-term CSHI, and after 18 months, the subjective health status was still improved. The included patients were all classified as “difficult-to-treat,” and when entering the study, all patients had one or more comorbidities. In the pilot study by Løvås and Husebye [11], CSHI improved HRQoL measurements, although only statistically significant for physical functioning and vitality subscales. Patients in the present study were closely monitored by physicians, especially around the change of treatment to CSHI. It is a risk that the reported subjective improvement in quality of life found in this and other studies could be related to closer follow-up by the physicians rather than being caused by the treatment itself. In addition, the fact that patients are suffering from other conditions than AI makes it harder to differentiate between the causes of the experienced changes in symptoms. As an example, two patients suffering from a chronic pain condition, and three patients were diagnosed with type 1 diabetes. It is reasonable to suggest that these conditions could interfere with the quality of life. Ultimately, randomized controlled trials should determine the efficacy of CSHI as a treatment alternative for patients with AI. However, the fact that the patients in our study felt better after the introduction of CSHI was also reflected in their number of hospitalizations. After a change of treatment to CSHI, the number of hospital admissions per year due to the adrenal crisis was halved. This might be explained by a better handling of cortisol deficit and thus avoidance of hospital admission. The duration of treatment with CSHI varies among the patients in this study. Three out of nine patients started CSHI within the last year. It is reasonable to believe that the reduction in hospitalizations per year due to adrenal crisis would be more pronounced with a longer follow-up period since some hospital admissions with adrenal crisis appear around the change of treatment to CSHI that does require more technical skills. In the study by Khanna et al. [18], the CSHI system also reduced the number of hospital admissions in 2 out of 3 patients, as well as days spent in the hospital, and thus lowered the estimated cost of care per patient. The authors argued that CSHI was cost-effective compared to standard OHC treatment if the candidates are selected carefully (e.g., if oral administration is unreliable or ineffective). Before changing to CSHI, the included patients had frequently been hospitalized due to treatment failure and adrenal crisis, which was also the case with several patients in our case series. Symptoms of cortisol deficit include nausea and vomiting, which makes oral delivery of cortisol challenging, especially when higher doses are needed. Eight out of nine patients in this cohort found it easier to handle symptoms of ensuing adrenal crisis after they got the pump. Oral treatment is of cause less invasive, and several patients answered that they found treatment with CSHI more troublesome compared to treatment with OHC (6 out of 9). Despite this, all patients preferred CSHI over OHC due to the positive effect on their general well-being. In the study by Khanna et al. [18], the treatment failure on OHC also occurred because of gastrointestinal disease and vomiting. Not only did the change of treatment to CSHI reduce the number of hospital admissions in the three patients, but it also improved the subjective well-being of the patients (retrospectively). This emphasizes the thought that CSHI may reduce the number of hospital admissions and improve the quality of life in selected patients experiencing severe symptoms of cortisol deficit on OHC. We performed a rough calculation comparing the cost of pump, medication, and utensils, comparing this with the cost of acute hospitalizations, which most patients experienced many times, and showed that avoidance of just 1-2 such acute hospitalization per year could more than cover the cost of CSHI. Needless to say, such cost-effect calculations should be performed in more depth by health economics researchers that have more experience with also including quality of life, and other costs.

There is a lack of larger trials comparing the effect of CSHI with conventional OHC, and besides the study by Björnsdottir et al. [13], only two randomized clinical trials (RCTs) have been conducted. The first was a multicenter, crossover, and open-labeled RCT conducted by Oksnes et al. [24] in 2014. The study showed promising results when 3 months of treatment with thrice-daily OHC was compared to CSHI in 32 patients with AD. The primary outcome was ACTH levels, and CSHI yielded a normalization of morning ACTH and serum cortisol, in contrast to low serum cortisol levels found on OHC, thus re-establishing the circadian cortisol rhythm. In addition, AddiQoL, and certain subscales of more generic questionnaires, improved with CSHI, although the effect on the HRQoL scores was not as obvious as in the pilot study conducted by Løvås et al. [11]. Patients in the pilot study were included due to poor functioning on standard OHC, while in the RCT study by Oksnes et al. [24], patients were included regardless of their health status. The more significant rise in AddiQol scores in the pilot study indicates that CSHI is more beneficial for patients with a poorly controlled disease on OHC.

Gagliardi et al. [25] also published a randomized double-blind placebo-controlled crossover trial in 2014. Patients received CSHI plus oral placebo, or OHC plus placebo infusion, in 4 weeks in a random order, with a 2-week washout period. Ten patients with AD completed the trial. The primary outcome was subjective health status measured with several different health-related questionnaires. The study casted some doubt on the potential benefit from CSHI, as they found no significant change in subjective health status when OHC was compared to CSHI. However, the included patients had a better baseline subjective health status compared with other AD cohorts. The low statistical power was another limitation, as only 10 patients completed the study. Furthermore, it is possible that 4 weeks of treatment was not enough time to detect changes in the subjective health status. Overall, results from former studies indicate that AI patients with a bad baseline status benefit from a change of treatment from OHC to CSHI, but this might not be the case for well-functioning patients, and results from the RCTs emphasize the importance of selecting the right patients for CSHI.

The present study is limited by the nature of it being a case series. To infer a more precise cause-effect relationship, RCTs are needed, but since AI is a rare disease RCTs can be difficult to perform due to a lack of patients. Thus, we do believe this study offers useful knowledge for elucidating the use of CSHI. Patients in this study experienced symptoms of inappropriate GC replacement on OHC and were strictly selected for treatment with CSHI. In general, findings in case reports provide little basis for generalization; however, this was not just a single case. Several AI patients with a poorly controlled disease have been found to benefit from CSHI. This strengthens the evidence for the use of CSHI in selected patients and it emphasizes the demand for further research.

We conclude that the change of treatment from oral GCs to CSHI resulted in better disease control on a lower daily dose of GCs and improved quality of life. In addition, the dose of GC was reduced, and likewise was the number of hospital admissions. These results provide further evidence for the use of CSHI in treating selected patients with poorly controlled AI. However, the sparse literature calls for larger studies to investigate both short-and long-term health benefits offered by CSHI in poor functioning patients with AI. Although the recent studies are promising, and CSHI seems to offer a more physiological cortisol replacement than OHC, in addition to a reduction in the daily GC exposure, a bigger randomized placebo-controlled trial on selected patients with poorly controlled AI is needed. Focus should center around effects on HRQoL, hospitalization, and signs of under or overreplacement with GC.

## Figures and Tables

**Table 1:** Patient characteristics.

Patient	Sex^a^	Age	BMI	Diagnosis	CSHI start	Comorbidity
1	M	47	36.5	Morphine induced combined pituitary hormone deficiency	30/9-21	Chronic pain condition
						Hypertension

2	F	45	20.6	Sheehan's syndrome	17/6-21	

3	M	58	38.8	Steroid induced adrenal insufficiency	20/5-21	Chronic pain condition

4	F	52	39.9	Addison's disease	22/11-18	Type 1 diabetes
						Hypothyroidism
						Ulcerative colitis

5	F	36	20.3	Addison's disease	01/11-09	Type 1 diabetes
						Epilepsy

6	F	48	23	Steroid induced secondary adrenal insufficiency	18/6-20	

7	M	55	29.1	Addison's disease	22/10-18	Type 1 diabetes

8	M	44	26.9	Addison's disease	1/10-20	Idiopathic intracranial hypertension

9	F	55	42.4	Congenital adrenal hyperplasia	8/5-19	Ulcerative colitis

^a^M:  male and F: female.

**Table 2:** Number of hospital admissions due to adrenal crisis per year before and on continuous subcutaneous hydrocortisone infusion.

Hospital admissions per year			
Patient	Before	After	Change
1	0.0	0.0	0.0
2	0.0	0.0	0.0
3	10.6	6.0	−4.6
4	7.3	5.5	−1.8
5	1.6	0.0	−1.6
6	0.0	0.0	0.0
7	0.0	0.0	0.0
8	1.7	0.0	−1.7
9	2.3	0.6	−1.7
Average	2.6	1.3	−1.3

**Table 3:** Body mass index before and after the introduction of continuous subcutaneous hydrocortisone infusion.

Body mass index				
Patient	Duration of CSHI, (months)	BMI before CSHI initiation, (kg/m^2^)	BMI after CSHI initiation, (kg/m^2^)	BMI change, (kg/m^2^)
1	4	27.1	26.2	−0.9
2	8	21.8	20.6	−1.2
3	9	40.6	38.8	−1.8
4	38	39.2	39.9	+0.7
5	147	?	20.3	
6	21	23	23	0
7	39	33.9	29.1	−4.8
8	16	29.4	26.9	−2.5
9	33	38.1	42.4	+4.3
Average	35	31.6	31.1	−0.5

CSHI:  continuous subcutaneous hydrocortisone infusions. BMI:  weight (kg)/(height (m))^2^.

**Table 4:** Results from the quality of life questionnaire.

	Number of patients^a^
Finds it easier to overcome everyday activities	8
Feels more rested in the morning	7
Has more energy during the day	7
Sleeps better at night	3
Is in a more positive mood	8
Increased sex drive/a better sex life	4
Easier to concentrate/feels cognitively sharper	5
Has more surplus for social activities	4
Less headache	4
Less abdominal pain and nausea	8
Less muscle pain	3
Less infections	0
Prefers the pump over the tablets	9
Finds it easier to handle a crisis	9
Finds the pump more troublesome	6
Has psychological problems with bearing a pump	2

^a^Out of 9 patients.

**Table 5:** Changes in subjective well-being.

How are you today compared to before you got the pump?^a^			
Patient number	Before	After	Change
1	1	10	+9
2	4	8	+4
3	1	6	+5
4	1	4	+3
5	2	10	+8
6	3	9	+6
7	1	10	+9
8	1	9	+8
9	1	10	+9
Average	1.75	8.25	+6.8

^a^1 is bad and 10 is good.

## Data Availability

The data that supports the findings of this study are available in the supplementary material of this article.

## Supplementary Materials

Supplementary Materials File 1: History of hospital admissions from patient records. Hospital admissions are divided into visits to the emergency department, hospital admissions without Addison crisis, and hospital admission with Addison crisis. File 2: Questionnaire used to interview the patients. The questionnaire focus on, how CSHI has effected life of the patients. File 3: Information regarding the patients' medical treatment before changing to CSHI.
