# Supplementary material for: Patients with Hypocortisolism Treated with Continuous Subcutaneous Hydrocortisone Infusion (CSHI): An Option for Poorly Controlled Patients
Source: Int J Endocrinol. 2023 Mar 20;2023:5315059. doi: 10.1155/2023/5315059 (PMC10042637; doi:10.1155/2023/5315059)
Supplement: Supplementary Materials — File 1: History of hospital admissions from patient records. Hospital admissions are divided into visits to the emergency department, hospital admissions without Addison crisis, and hospital admission with Addison crisis. File 2: Questionnaire used to interview the patients. The questionnaire focus on, how CSHI has effected life of the patients. File 3: Information regarding the patients' medical treatment before changing to CSHI. [file 5315059.f1.docx]

# Supplementary information 1

History of hospital admissions from patient records. Hospital admissions are divided into visits at the emergency department, hospital admissions without Addison crisis (AC) and hospital admission with AC. Admissions with adrenal crisis were counted and calculated as total number of admissions in the full calendar year before switch to CSHI plus the months of the year until the switch, divided by number of months, and multiplied by 12, thus representing number of admissions the year before switch to CSHI. Likewise, after switch to CSHI, hospital admissions with adrenal crisis were counted in the remaining months of the year after the switch plus one full calendar year (or less if the switch was done recently at the time of medical report review), divided by number of months, and multiplied by 12, representing number of admissions the year after the switch.

**Patient 1**

|  | Emergency  department | Hospital  admissions (no AC) | Hospital  admissions (with AC) | Total |
| --- | --- | --- | --- | --- |
| 2022 | 0 | 0 | 0 | 0 |
| 2021 after | 0 | 0 | 0 | 0 |
| 2021 before | 2 | 0 | 0 | 2 |
| 2020 | 2 | 0 | 0 | 2 |

**Patient 2**

|  | Emergency  department | Hospital  admissions (no AC) | Hospital  admissions (AC) | Total |
| --- | --- | --- | --- | --- |
| 2013-2022 | 0 | 0 | 0 | 0 |
| 2012 | 1 | 0 | 0 | 1 |

**Patient 3**

|  | Emergency  department | Hospital  admissions (no AC) | Hospital  admissions (AC) | Total |
| --- | --- | --- | --- | --- |
| 2022 | 0 | 0 | 1 | 1 |
| 2021 after | 0 | 1 | 3 | 4 |
| 2021 before | 0 | 1 | 3 | 4 |
| 2020 | 1 | 2 | 12 | 15 |
| 2019 | 0 | 0 | 4 | 4 |

**Patient 4**

|  | Emergency  department | Hospital  admissions (no AC) | Hospital  admissions (AC) | Total |
| --- | --- | --- | --- | --- |
| 2022 | 0 | 1 | 0 | 1 |
| 2021 | 0 | 8 | 0 | 8 |
| 2020 | 0 | 8 | 1 | 9 |
| 2019 | 0 | 7 | 6 | 13 |
| 2018 after | 0 | 3 | 0 | 3 |
| 2018 before | 0 | 3 | 7 | 10 |
| 2017 | 0 | 2 | 7 | 9 |
| 2016 | 0 | 1 | 9 | 10 |
| 2015 | 0 | 0 | 4 | 4 |
| 2014 | 0 | 0 | 2 | 2 |
| 2013 | 0 | 0 | 0 | 0 |
| 2012 | 1 | 0 | 1 | 2 |
| 2011 | 0 | 0 | 0 | 0 |
| 2010 | 0 | 0 | 0 | 0 |
| 2009 | 0 | 0 | 3 | 3 |
| 2008 | 0 | 0 | 0 | 0 |
| 2007 | 0 | 0 | 0 | 0 |
| 2006 | 0 | 0 | 1 | 1 |
| 2005 | 0 | 0 | 1 | 1 |

**Patient 5**

|  | Emergency  department | Hospital  admissions (no AC) | Hospital  admissions (AC) | Total |
| --- | --- | --- | --- | --- |
| 2022 | 1 | 0 | 0 | 1 |
| 2021 | 0 | 0 | 0 | 0 |
| 2020 | 0 | 0 | 0 | 0 |
| 2019 | 0 | 1 | 0 | 1 |
| 2018 | 0 | 1 | 0 | 1 |
| 2017 | 0 | 2 | 0 | 2 |
| 2016 | 0 | 1 | 0 | 1 |
| 2015 | 0 | 0 | 0 | 0 |
| 2014 | 0 | 2 | 0 | 2 |
| 2013 | 1 | 2 | 0 | 3 |
| 2012 | 0 | 1 | 0 | 1 |
| 2011 | 0 | 0 | 0 | 0 |
| 2010 | 0 | 0 | 0 | 0 |
| 2009 after | 0 | 0 | 0 | 0 |
| 2009 before | 0 | 3 | 0 | 3 |
| 2008 | 0 | 2 | 3 | 5 |
| 2007 | 1 | 0 | 2 | 3 |

**Patient 6**

|  | Emergency  department | Hospital  admissions (no AC) | Hospital  admissions (with AC) | Total |
| --- | --- | --- | --- | --- |
| 2022 | 0 | 0 | 0 | 0 |
| 2021 | 2 | 0 | 0 | 2 |
| 2020 after | 1 | 0 | 0 | 1 |
| 2020 before | 0 | 0 | 0 | 0 |
| 2019 | 0 | 0 | 0 | 0 |
| 2018 | 0 | 0 | 1 | 1 |

**Patient 7**

|  | Emergency  department | Hospital  admissions (no AC) | Hospital  admissions (with AC) | Total |
| --- | --- | --- | --- | --- |
| 2022 | 0 | 0 | 0 | 0 |
| 2021 | 2 | 0 | 0 | 2 |
| 2020 | 0 | 0 | 0 | 0 |
| 2019 | 0 | 0 | 0 | 0 |
| 2018 after | 0 | 0 | 0 | 0 |
| 2018 before | 0 | 0 | 0 | 0 |
| 2017 | 0 | 0 | 0 | 0 |
| 2016 | 0 | 0 | 0 | 0 |
| 2015 | 1 | 0 | 2 | 3 |

**Patient 8**

|  | Emergency  department | Hospital  admissions (no AC) | Hospital  admissions (AC) | Total |
| --- | --- | --- | --- | --- |
| 2022 | 0 | 0 | 0 | 0 |
| 2021 | 2 | 1 | 0 | 3 |
| 2020 after | 0 | 0 | 0 | 0 |
| 2020 before | 1 | 0 | 0 | 1 |
| 2019 | 1 | 1 | 3 | 5 |
| 2018 | 0 | 0 | 1 | 1 |
| 2017 | 0 | 0 | 3 | 3 |

**Patient 9**

|  | Emergency  department | Hospital  admissions (no AC) | Hospital  admissions (with AC) | Total |
| --- | --- | --- | --- | --- |
| 2022 | 0 | 0 | 0 | 0 |
| 2021 | 0 | 0 | 0 | 0 |
| 2020 | 0 | 0 | 0 | 0 |
| 2019 after | 0 | 0 | 1 | 1 |
| 2019 before | 0 | 0 | 1 | 1 |
| 2018 | 3 | 0 | 2 | 5 |
| 2017 | 0 | 1 | 8 | 9 |
| 2016 | 5 | 8 | 12 | 25 |
| 2015 | 1 | 8 | 9 | 18 |
| 2014 | 0 | 1 | 2 | 3 |
| 2013 | 1 | 4 | 5 | 10 |
| 2012 | 3 | 1 | 0 | 4 |
| 2011 | 0 | 0 | 1 | 1 |

# Supplementary information 2

The questionnaire presented below is written in Danish but if requested it can be translated.

Spørgeskema

Indledende spørgsmål:

Hvad var din baggrund for at søge at komme i pumpebehandling?

Hvordan oplevede du skiftet fra tablet til pumpebehandling?

Hvor mange tilfælde af Addisonkrise har du oplevet efter skiftet?
*Supplerende: Hvor mange har været indlæggelseskrævende*

Hvor mange tilfælde af Addisonkriser havde du før skiftet?

*Supplerende: Hvor ofte, var du indlagt?*

Er det blevet nemmere/sværere at håndtere en Addison krise?

Supplerende spørgsmål:

**Hverdagen**

Har din dagligdag/daglige aktiviteter ændret sig?

*Supplerende:* Er der aktiviteter du kan udføre nu, som du ikke kunne før?

Har dit energiniveau ændret sig?

*Supplerende: Er du mere eller mindre træt i løbet af dagen?*

Har du oplevet ændringer i, hvordan du sover om natten?

*Supplerende: Ændringer i, hvor frisk du er, når du vågner op?*

**Helbred**

Oplever du, at du er mere eller mindre syg?

Specifikke symptomer før og efter skift i behandling:

- Hovedpine:
- Kvalme- og mavesmerter:
- Muskel- og ledsmerter og rygsmerter:

**Generelt**

Har dit humør ændret sig?

Har dit sexliv/din sexlyst ændret sig?

Har din evne til at koncentrere sig ændret sig?

Har din appetit ændret sig?

Har din vægt ændret sig?

Afsluttende spørgsmål:

Foretrækker du pumpe-behandlingen frem for tablet-behandlingen?

På en skala fra 1 til 10, hvordan havde du det før, du fik pumpen, og hvordan har du det nu?

Hvilke ulemper/bivirkninger vil du fremhæve/har du oplevet ved pumpebehandlingen?

Ud over de ting vi har snakket om, har du så ellers noget at tilføje i forhold til dit forløb med skift til pumpe-behandlingen?

# Supplementary information 3

Information regarding the patients´ treatment before changing to CSHI.

| Patient | Daily treatment | Hydrocortisone equivalent (mg/day) |
| --- | --- | --- |
| 1 | Plenadren: 20 mg + 20 mg + 20 mg  Hydrocortisone: 10 mg + 10 mg | 80 |
| 2 | Plenadren: 20 mg  Hydrocortisone: 5 mg | 25 |
| 3 | Plenadren: 40 mg | 40 |
| 4 | Hydrocortisone: 20 mg + 10 mg + 10 mg | 40 |
| 5 | Hydrocortisone: 20 mg + 20 mg + 20 mg | 60 |
| 6 | Plenadren: 20 mg  Hydrocortisone: 5 mg | 25 |
| 7 | Plenadren: 30 mg  Hydrocortisone: 2.5 mg | 32.5 |
| 8 | Plenadren: 20 mg  Hydrocortisone: 25 mg + 20 mg | 65 |
| 9 | Hydrocortisone: 30 mg + 20 mg + 10 mg | 60 |
